# A Comparative Study on the Melt Crystallization of Biodegradable Poly(butylene succinate-*co*-terephthalate) and Poly(butylene adipate-*co*-terephthalate) Copolyesters

**DOI:** 10.3390/polym16172445

**Published:** 2024-08-29

**Authors:** Pengkai Qin, Linbo Wu

**Affiliations:** 1 Key Laboratory of Biomass Chemical Engineering of Ministry of Education, College of Chemical and Biological Engineering, Zhejiang University, Hangzhou 310058, China; qinpk@zju.edu.cn; 2 State Key Laboratory of Chemical Engineering, College of Chemical and Biological Engineering, Zhejiang University, Hangzhou 310058, China

**Keywords:** biodegradable polymers, biobased polymers, aliphatic-aromatic copolyesters, melt crystallization, transparency, mechanical property, poly(butylene succinate-*co*-terephthalate), poly(butylene adipate-*co*-terephthalate)

## Abstract

As an important biodegradable and partially biobased copolyester, poly(butylene succinate-*co*-terephthalate) (PBST) possesses comparable thermal and mechanical properties and superior gas barrier performance when compared with poly(butylene adipate-*co*-terephthalate) (PBAT), but it was found to display poorer melt processability during pelletizing and injection molding. To make clear its melt crystallization behavior under rapid cooling, PBST_48_ and PBST_44_ were synthesized, and their melt crystallization was investigated comparatively with PBAT_48_. PBST_48_ showed a PBAT_48_-comparable melt crystallization performance at a cooling rate of 10 °C/min or at isothermal conditions, but it showed a melt crystallization ability at a cooling rate of 40 °C/min which was clearly poorer. PBST_44_, which has the same mass composition as PBAT_48_, completely lost its melt crystallization ability under the rapid cooling. The weaker chain mobility of PBST, resulting from its shorter succinate moiety, is responsible for its inferior melt crystallization ability and processability. In comparison with PBAT_48_, PBST_48_ displayed higher tensile modulus, and both PBST_48_ and PBST_44_ showed higher light transmittance. The findings in this study deepen the understanding of PBST’s properties and will be of guiding significance for improving PBST’s processability and application development.

## 1. Introduction

Aliphatic-aromatic copolyesters refer to a class of polyesters synthesized from an aliphatic diol, an aliphatic *α*,*ω*-diacid, and an aromatic diacid. Such copolyesters can be biodegradable within a suitable composition range [1,2,3,4]. Among them, only the copolyesters synthesized from 1,4-butanediol (BDO), terephthalic acid (TPA), and adipic acid (AA) or succinic acid (SA) have been commercialized to date. Poly(butylene adipate-*co*-terephthalate) (PBAT) was commercialized in the mid-1990s and gradually found applications in various disposable film products like shopping bags, express packages, and mulch film, due to its biodegradability, thermoplastic processability, well-balanced thermal/mechanical properties [5,6,7], and relatively low cost. It is one of the most widely used biodegradable polymers today. Researchers on the modification and applications of PBAT are still very active in recent years [8,9,10,11,12,13,14].

Poly(butylene succinate-*co*-terephthalate) (PBST) is another biodegradable aliphatic-aromatic copolyester, with a structure and properties similar to PBAT [15,16,17,18]. After extensive studies on synthetic technology [19,20,21] and process optimization [22,23], PBST was industrialized at Sinopec Yizheng Chemical Fiber Co., Ltd., Yizheng, China in 2020. It is a partially biobased polymer because SA is a biobased monomer [24,25]. In fact, biobased SA has been commercially produced at a cost comparable to petroleum-based SA. As the cost of biobased succinic acid gradually declines, the cost of PBST will become increasingly competitive, although it is currently higher than that of PBAT. Furthermore, we found recently that PBST displays 3–4 times more oxygen and carbon dioxide and a 1.6 times stronger water vapor barrier performance when compared with PBAT. This demonstrates that the two fewer methylene groups in the succinate moiety of PBST result in a smaller free volume fraction and a slower gas diffusion [26]. The advantages of PBST’s gas barrier have been confirmed by other subsequent reports [27]. The biobased nature and higher gas barrier make PBST more promising in demanding applications such as food package materials. In fact, the R&D of a variety of differentiated products based on PBST has been reported recently [28,29,30,31,32,33].

Although PBST has advantages in sustainability and the gas barrier, its melt processability seems inferior to that of PBAT. During PBST’s industrial development, it was found that it is harder to cool it down in time for pelleting after melt polycondensation or for demolding in injection molding. In comparison, PBAT is clearly easier to pelletize and inject under the same conditions. The poorer melt processability of PBST suggests inferior melt crystallizability. However, in most reports on PBST crystallization in the literature [16,34,35], PBST displayed melt crystallization behavior similar to PBAT, with the exception of the findings of Heidarzadeh et al. [36]. Heidarzadeh et al. reported that PBST melt crystallization demonstrates a higher degree of super-cooling than PBAT. As the cooling rate (mostly, 10 °C/min) used in these DSC studies of PBST [16,34,35] is much slower than that in the real melt-processing process, these DSC results do not reflect the real melt crystallizability of PBST in the industrial process. Therefore, it is necessary to evaluate the melt crystallizability at a rapid cooling rate. On the other hand, the fact that PBST possesses a significantly higher gas barrier than PBAT [26] reminds us that the tiny difference in chain structure between them may also result in a clear difference in melt crystallization and even in some crystallization-related macroscopic properties. Although the physical and mechanical properties of PBST and PBAT have been extensively studied [5,6,7,15,16,17,18,19,20,21], a comparative study is still missing.

In this study, PBAT_48_, PBST_48,_ and PBST_44_ copolyesters with the same molar (48 mol% BT) or mass (50 wt% BT) composition as commercial PBAT resin were synthesized, and their melt crystallization behaviors at normal cooling, rapid cooling, and isothermal condition were studied comparatively. Their light transmittance performance and tensile properties were also studied.

## 2. Experimental Section

### 2.1. Materials

One PBAT (PBAT_48_, the subscript means the molar percentage of butylene terephthalate (BT) unit) and two PBST (PBST_44_, PBST_48_) samples were synthesized in a 2.5 L stainless reactor from terephthalic acid (99%, Hengyi PetroChem., Co., Hangzhou, China), 1,4-butanediol (99.5%, Mitsubishi Chem. Co., Ltd., Ningbo, China), succinic acid (99%, Anhui Sanxin Chem. Co., Chizhou, China) or adipic acid (99%, Liaoyang Petrochem. Co., Liaoyang, China) using tetrabutoxyl titanium (TBT, 99.8%, J&K Chem., Beijing, China) as a catalyst. First, esterification was conducted at a diol/diacid molar ratio of 2/1 and 220 °C for about 3.5 h in the presence of 0.05 mol% TBT based on diacid until over 95 wt% water was stilled out. Then, an additional 0.05 mol% TBT was added, and the pressure was slowly reduced. The temperature was gradually raised to 250 °C over 45 min. Finally, melt polycondensation was then carried out at 250 °C and 20–100 Pa for about 3 h. 

The copolyesters were melt-processed by a heat press (GT-7014-A50C, Taiwan, China) under about 15 MPa at 165 °C to prepare film samples with a thickness of about 400 μm. The films were directly used for WAXD observation and optical property measurement. Dumbbell-shaped standard specimens (2 × 25 × 0.4 mm^3^) were prepared from the films by a cutter and used for the tensile test. 

### 2.2. Characterization

The copolyesters (0.1250 g) were dissolved in chloroform. The resulting solution (0.005 g/mL) was used for an intrinsic viscosity (IV) measurement at 25 °C using a IVS300 semi-automatic viscometer tester (Hangzhou Zhongwang Co., Hangzhou, China) equipped with an Ubbelohde viscometer (inner diameter 0.36 mm). ^1^H NMR spectra were recorded with a Bruker AC-80 spectroscopy instrument (400 M). Deuterated chloroform was used as the solvent and tetramethylsilane was used as the internal reference. The pulse sequence, temperature, and pulse duration were one pulse, 25 °C, and 1 s, respectively.

Thermal transition behaviors of PBAT and PBST were recorded with differential scanning calorimetry (DSC, Q200, TA Instrument Co., New Castle, DE, USA) under nitrogen flow. For nonisothermal crystallization and melting, the samples (6–8 mg) were heated at 10 °C/min from room temperature to 220 °C, kept at this temperature for 5 min, then cooled at 10 °C/min or 40 °C/min to −80 °C, kept at this temperature for another 5 min, and finally heated to 220 °C at 10 °C/min. For the isothermal crystallization and melting, the samples were heated at 10 °C/min from room temperature to 220 °C, kept at this temperature for 5 min, then quenched to the predetermined melt crystallization temperature (*T*_c_), kept at this temperature for 30 min, then cooled at 10 °C/min to −80 °C, and finally heated to 220 °C at 10 °C/min.

Wide-angle X-ray diffraction (WAXD) patterns of PBAT and PBST were recorded with the X-ray diffractometer (PANalytical B.V., X-pert-Powder, Almelo, The Netherlands) with a CuKα radiation (1.54 Å), working at 40 KV and 40 mA. The sample was scanned from 2θ = 5° to 2θ = 80° with a step size of 0.026° and an acquisition time of 30 s per step.

The transmittance and haze of the copolyesters were determined using a CS-821 N desktop spectrophotometric colorimeter (Hangzhou CHNSpec Technology Co. Ltd., Hangzhou, China). All of the tests were carried out in the visible wavelength range 400–800 nm. 

The tensile properties of the copolyesters were measured with a Zwick/Roell Z020 (Zwick Co., Ulm, Germany) universal testing machine at a tensile speed of 50 mm/min. The value of the load cell and preload were 500 N and 0 N, respectively. All of the specimens were kept at 25 °C and 50% relative humidity for at least 48 h before testing. At least five specimens were tested for each sample.

## 3. Results and Discussion

As the BT unit content in commercial PBAT is usually about 47–48 mol% or 50–51 wt%, one PBAT (PBAT_48_) and two PBST (PBST_44_ and PBST_48_) samples with the same molar (~48 mol%, PBAT_48_ and PBST_48_) or mass percentage (~50 wt%, PBAT_48_ and PBST_44_) of BT unit were synthesized and used for this study. The ^1^H NMR characterization and calculation of the chemical composition, average sequence length, and randomness degree are listed in the supporting information (Figure S1). The copolymer molar and mass compositions, the average sequence length, the randomness degree, and the intrinsic viscosity of the copolyesters are listed in Table 1. Random copolyesters with high-enough intrinsic viscosity (>1.0 dL/g, chloroform as solvent) and expected copolymer composition were successfully synthesized.

**Figure 1:**
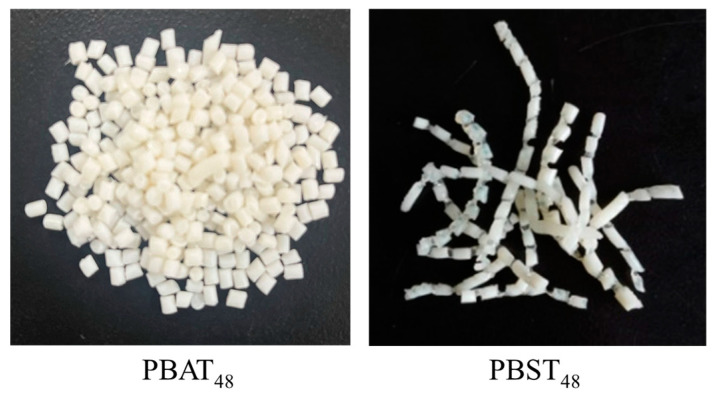
Picture of pelletized samples of PBAT_48_ and PBST_48_.

When the resin melt was discharged out of the bottom outlet of the 2.5 L reactor with the aid of nitrogen pressure, stretched into thin strips and cooled by tap-water in a 2-m long tank, and then cut into pellets by a pelletizer, it was found that PBAT_48_ was easily cut off but that PBST_48_ was not. The PBST pellets were connecting with each other, as shown in Figure 1. PBST_44_ was too soft after water cooling and so more difficult than PBST_48_ to be cut off under the same condition. The result implies that the melt crystallization of these copolyesters under rapid cooling was quite different. Although there are extensive studies on the melt crystallization of PBST [16,34,35] at a conventional cooling rate, research on melt crystallization in the case of rapid cooling is still lacking. So, the isothermal melt crystallization and the nonisothermal melt crystallization of PBAT and PBST at rapid and conventional cooling rates are comparatively investigated in this work.

### 3.1. Nonisothermal Melt Crystallization and Melting

**Figure 2:**
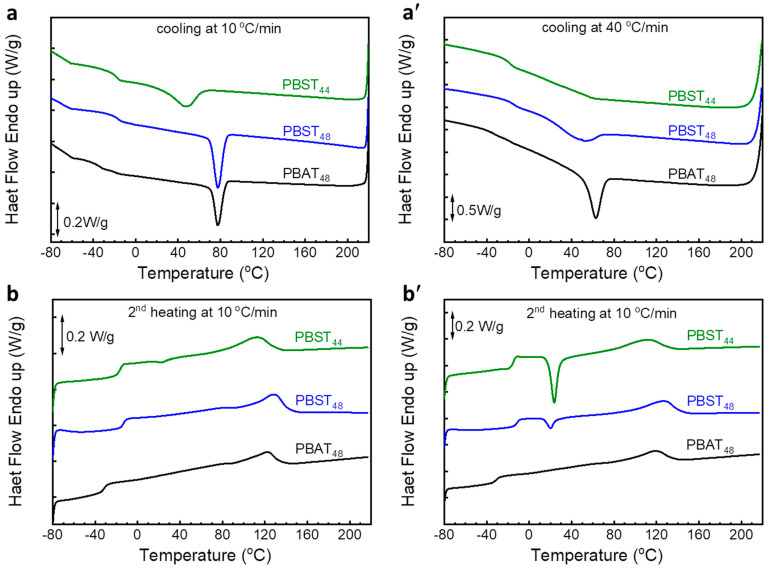
DSC cooling ((**a**): 10 °C/min; (**a′**): 40 °C/min) and 2nd heating ((**b**,**b′**): 10 °C/min) scan curves of PBAT_48_, PBST_44_ and PBST_48_.

First, the thermal transition behaviors of PBAT_48_, PBST_48_, and PBST_44_ were studied with DSC at a cooling/heating rate of 10 °C/min, which is widely used in DSC studies of PBST in the literature [16,34,35] After erasing the heat history, all of the copolyesters showed melt crystallization during cooling at 10 °C/min (Figure 2a), then glass transition and melting occurred during the 2nd heating at 10 °C/min (Figure 2b). The melt crystallization and 2nd melting peaks are attributed to the BT sequence. The BA and BS sequences did not crystallize from melt under the testing condition. But melting peaks of BA and BS sequences were indeed observed in the 1st heating scan (see Figure S2 in supporting information). It has been reported that, although the BS crystal of PBST can not be detected by X-ray diffraction, it can indeed be formed slowly after long-time storage at room temperature, and therefore can be observed in the first DSC scan [35]. The results in Figure S2 provide evidence for a similar conclusion regarding the copolyesters in this study.

At the cooling rate of 10 °C/min, PBAT_48_ and PBST_48_ showed almost the same melt crystallization behavior, with almost the same temperature, enthalpy, and half period of melt crystallization (*T*_c_ 78 °C, Δ*H*_c_ 19.8–20.4 J/g, *t*_1/2_ 43–45 s, see Table 2). No cold crystallization was observed in the secondary heating curve, suggesting that the melt crystallization of them occurred sufficiently. In comparison with the thermal transition properties of commercial PBAT [37,38], the PBAT_48_ sample displayed almost the same transition temperatures (*T*_g_, *T*_c_, *T*_m_) but higher Δ*H*_c_ and Δ*H*_m_. The existence of branched or chain extender structures in the macromolecular chain of commercial PBAT may account for its lower transition enthalpies. In comparison, PBST_44_ clearly showed poorer melt crystallizability. The *T*_c_, Δ*H*_c_, and *t*_1/2_ of PBST_44_ were 47 °C, 17.3 J/g, and 98 s, respectively. Its melt crystallization took place at a higher supercooling degree at a much slower rate (*t*_1/2_ over 2 times). Due to insufficient melt crystallization, weak but still obvious cold crystallization (Δ*H*_cc_ = 1.2 J/g) can be observed during the 2nd heating. In summary, at the conventional DSC cooling rate of 10 °C/min, PBST_48_ has excellent melt crystallizability comparable to that of PBAT_48_ and better than that of commercial PBAT [37,38]. Its thermal transition properties are also consistent with those reported for PBST_45–50_ in the literature [16,34,35]. But the PBST_44_ with lower *ϕ*_BT_ but the same *ϕ*_w,BT_ shows significantly poorer crystallizability than PBAT_48_. It seems that the molar composition is the determining factor for the melt crystallization of these copolyesters.

As the cooling rate during practical melt processing is much faster than the conventional DSC cooling rate, the above DSC results do not represent the melt crystallization behavior under real processing conditions. To make clear the melt crystallization of PBST and PBAT under real processing conditions, the DSC cooling rate was raised to 40 °C/min (the maximum controllable cooling rate of the DSC instrument) and other conditions were kept unchanged. The DSC curves are shown in Figure 2a′,b′ and the results are summarized in Table 2 too.

**Figure 3:**
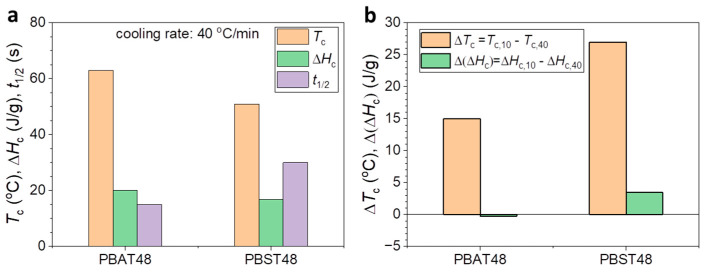
(**a**) The temperature, enthalpy, and half time of melt crystallization (*T*_c_, Δ*H*_c_, *t*_1/2_) of PBST_48_ and PBAT_48_ at cooling rate of 40 °C/min; (**b**) change in melt crystallization temperature (Δ*T*_c_) and enthalpy (Δ(Δ*H*_c_)) of PBST_48_ and PBAT_48_ between different cooling rates. The subscript “_10_” or “_40_” in *T*_c,10,_ *T*_c,40,_ Δ*H*_c,10,_ Δ*H*_c,40_ means the cooling rate, 10 or 40 °C/min.

In comparison with the results for the cooling rate of 10 °C/min, PBAT_48_ showed lower *T*_c_ (63 °C), shorter *t*_1/2_ (15 s), but constant Δ*H*_c_ (20.1 J/g) at a cooling rate of 40 °C/min, and no cold crystallization was observed during the second heating. Obviously, PBAT_48_ still manifested an excellent melt crystallization performance, though a higher supercooling degree was observed. Under the same cooling rate, the *T*_c_, Δ*H*_c,_ and *t*_1/2_ values of PBST_48_ were 51 °C, 16.9 J/g, and 30 s, respectively. The lower *T*_c_, smaller Δ*H*_c,,_ and longer *t*_1/2_ of PBST_48_ shown in Figure 3a indicate that it clearly has a poorer melt crystallizability than PBAT_48_ under rapid cooling conditions. An obvious cold crystallization (Δ*H*_cc_ = 4.7 J/g) in the 2nd heating was observed, indicating that the melt crystallization of PBST_48_ was insufficient. In order to compare the effect of the cooling rate on the melt crystallization of PBAT_48_ and PBST_48_ more clearly, the difference between the melt crystallization temperature and enthalpy (Δ*T*_c_ = *T*_c,10_ ࢤ *T*_c,40_, Δ (Δ*H*_c_) = Δ*H*_c,10_ ࢤ Δ*H*_c,40_) of PBAT_48_ and PBST_48_ are defined and compared in Figure 3b. It can be seen that the Δ*T*_c_ and Δ (Δ*H*_c_) values of PBST_48_ are clearly higher than those of PBAT_48_. This indicates that PBST_48_ was affected more remarkably than PBAT_48_ by the cooling rate in melt crystallization and exhibited weakened melt crystallizability at a cooling rate of 40 °C/min. As for PBST_44_, no obvious melt crystallization peak was observed during the rapid cooling, and there was a big cold crystallization peak (Δ*H*_cc_ 15.4 J/g) in the 2nd heating. This means that PBST_44_ almost completely lost its melt crystallization ability. The above results indicate that the melt crystallizability of PBST_48_ and PBST_44_ is clearly inferior to that of PBAT_48_ under rapid cooling, regardless of having the same molar or mass composition. This is the reason why PBST is more difficult to melt-process.

The inferior melt crystallization ability of PBST under rapid cooling can be attributed to its weaker chain mobility due to its shorter aliphatic diacid moiety (2 CH_2_ in succinate vs. 4 CH_2_ in adipate). The weaker chain mobility of PBST has been demonstrated by a smaller free volume fraction and a smaller gas diffusion coefficient in our previous study [26]. At a relatively slow cooling rate of 10 °C/min, the chain mobility of PBST_48_ is still high enough to meet the need of the chain rearrangement during melt crystallization. However, under rapid cooling conditions, PBST_48_ cannot provide enough chain mobility to rapidly rearrange its chain into the lattice. As a result, it needs a higher supercooling degree to start and a longer time to complete melt crystallization under rapid cooling. In comparison, PBAT has sufficient chain mobility to meet the demands of melt crystallization even under rapid cooling. 

### 3.2. Isothermal Crystallization and Melting Behavior

**Figure 4:**
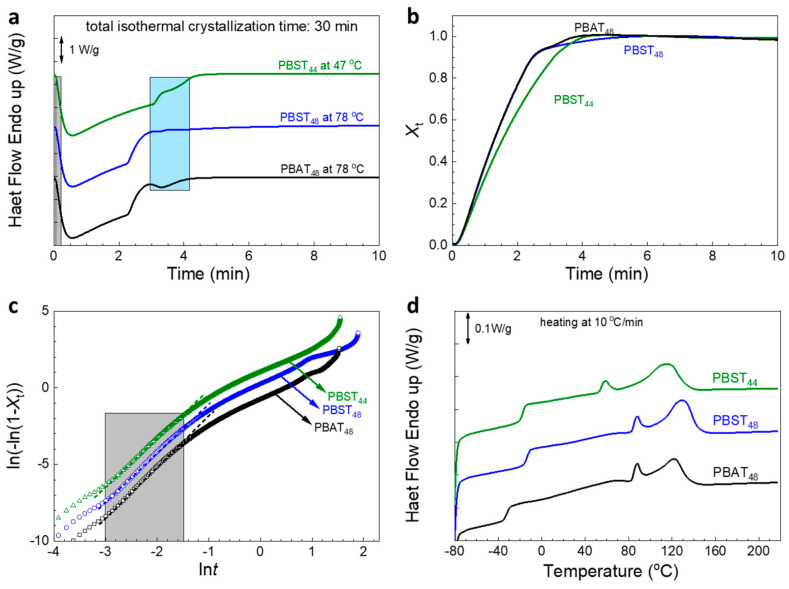
(**a**) Isothermal crystallization DSC curves; (**b**) time evolution of relative crystallinity (*X_t_*); (**c**) Avarami plot of ln(−ln(1 − *X_t_*)) vs. ln*t*. (**d**) 2nd heating (10 °C/min) curves after isothermal melt crystallization for 30 min and then cooling at 10 °C/min to −80 °C.

Li FX et al. studied the isothermal melt crystallization of PBST_70_ [39], but the isothermal melt crystallization of PBST_44–48_ has not been reported in the literature. Therefore, the isothermal melt crystallization behavior of PBAT_48_, PBST_48_, and PBST_44_ was further investigated in this study. The nonisothermal melt crystallization peak temperature at 10 °C/min cooling, namely, 78 °C, 78 °C, and 47 °C (see Table 2), was selected for the isothermal melt crystallization. After erasing the heat history, the melt was quenched to the isothermal temperature. As seen in Figure 4a, all of the three copolyesters displayed distinctive isothermal melt crystallization behaviors. First, two exothermic peaks appeared, and second, the peaks were very asymmetrical. Both peaks were attributed to the crystallization of the BT sequence. The smaller peak may correspond to the crystallization of the shorter BT sequences which could only crystallize more slowly to form immature crystals after the longer BT sequences had crystallized to form more perfect crystals.

From the thermal flow data in Figure 4a, the relative crystallinity (*X_t_*) was calculated with Equation (1) and plotted in Figure 4b. From the Avrami equation shown in Equation (2), the Avarami plots (Equation (3)) were made and shown in Figure 4c. The linear part in the grey area corresponding to the isothermal time range 0.05–0.22 min is used to calculate the crystallization rate constant *K* and the Avrami index *n* by linear fitting. The half time of isothermal melt crystallization, *t*_1/2_, is calculated from *n* and *K* with Equation (4). All the results are summarized in Table 3. The Avrami indexes *n* of all of the three copolyesters are close to three. The *K* and *t*_1/2_ values of PBST_48_ are equal to those of PBAT_48_, indicating that their isothermal melt crystallization rates are equal to each other. The *t*_1/2_ value of PBST_48_ (36.6 s) is obviously longer than that (11 s) reported for PBST_70_ at 80 °C [39], indicating much slower melt crystallization of PBST_48_ than PBST_70_. The *t*_1/2_ value of PBST_44_ is longer than that of PBST_48_ (0.68 min vs. 0.61 min), indicating a slower crystallization rate. These results are similar to the crystallization performance of non-isothermal melt crystallization at a cooling rate of 10 °C/min.

(1) $$X_{t}=\frac{\int_{0}^{t}(\Delta H_{c})dt}{\int_{0}^{\infty }(\Delta H_{c})dt}$$

(2) $$1-X_{t}=\exp(-Kt^{n})$$

(3) $$\ln\left[-\ln(1-X_{t})\right]=\ln K+n\ln t$$

(4) $$t_{1/2}={\left(\frac{\ln2}{K}\right)}^{1/n}$$

After 30 min of isothermal melt crystallization, the samples were cooled down further to −80 °C at 10 °C/min. Neither further melt crystallization during the cooling nor cold crystallization during the 2nd heating process were observed, indicating that they had sufficiently crystallized during the isothermal crystallization process. During the 2nd heating, they all displayed two melting peaks. The first smaller one represents the melting of the immature crystals formed at the later stage of isothermal melt crystallization, and the second/main peak represents the melting of the more perfect crystals formed at the earlier stage of isothermal melt crystallization. Although it is reported that the BS sequence in PBST_50_ can crystallize slowly at room temperature [35], the smaller peak in Figure 4d can not be regarded as the melting of BS or BA crystals. As the first *T*_m_ (88 °C) of PBAT_48_ is much higher than the equilibrium melting temperature of PBA (63 °C [40]), it is impossible to attribute the first melting peak of PBAT_48_ to the melting of BA crystal. Therefore, in can be deduced that the first peaks of PBST_48_ and PBST_44_ are not the melting of BS crystals.

The thermal transition properties during the 2nd heating are summarized in Table 4. For PBAT_48_, it can be seen that, although the Δ*H*_m2_ value (16.2 J/g) is lower, the total Δ*H*_m_ value (Δ*H*_m,sum_ = Δ*H*_m1_ + Δ*H*_m1_ = 18.8 J/g) is almost the same as the Δ*H*_m_ value (19.2 J/g) after nonisothermal melt crystallization at 10 °C/min (see Table 2). This result supports the conclusion that PBAT_48_ has strong melt crystallizability. In other words, the BT sequences in PBAT_48_ crystallized sufficiently during 10 °C/min cooling; as a result, the Δ*H*_m,sum_ could not be further increased by the annealing. But for PBST_48_ and PBST_44_, the Δ*H*_m,sum_ values (21.9, 20.1 J/g) are clearly higher than those values (19.7, 18.8 J/g) after nonisothermal melt crystallization at 10 °C/min (see Table 2), indicating that the two PBST samples did not crystallize sufficiently during the 10 °C/min cooling, and therefore the melt enthalpies were further increased after annealing. On the other hand, the two PBST samples displayed higher Δ*H*_m,sum_ values after isothermal melt crystallization than PBAT_48,_ regardless of weaker nonisothermal melt crystallizability. In other words, they can have slightly higher crystallinity than PBAT_48_.

### 3.3. Light Transmittance Performance

PBAT film is often translucent. But we find PBST film always displays better light transmittance performance than PBAT film. The light transmittance and haze of PBAT_48_, PBST_48_, and PBST_44_ films with thickness of ca. 425 μm were measured and compared in Table 5. It can be seen that all the films display almost the same haze but different transmittance in such an order: PBAT_48_ < PBST_48_ < PBST_44_. The difference in light transmittance might be related to their difference in crystallization and in the formed microscopic crystal morphology. The films were melt-processed by heat press at 165 °C and then cooled with cooling water. Due to the stronger chain mobility of PBAT_48_, it can be inferred that its crystals probably grew faster to form crystals with a bigger size than PBST_48_ and PBST_44_. For PBST_48_ and PBST_44_, it was difficult to finish the melt crystallization quickly under such a rapid cooling process, so the crystallization occurred mostly at lower temperatures. As lower temperature is known to favor nucleation over crystal growth, PBST_48_ and PBST_44_ crystals would have smaller size and consequently higher transmittance than PBAT_48_. However, the authors failed to demonstrate this point using polarized light optical microscopy (POM). Although clear spherulites can be observed for commercial PBAT [41] and PBST_70_ [39], there is in fact no report of the spherulite observation of PBST_44–48_ in the literature. In Lee SH et al.’s report, they discuss how PBST_10–20_ can form clear spherulites during a supercooling of 30 °C, but the spherulites structure of PBST_30_ is not clear and the typical Maltese-cross is not clearly seen, and for PBST_40_, no spherulite can be observed at all [18]. Zheng C et al. [35] demonstrated the existence of PBST_50_ spherulites with POM, but the spherulite boundary was blurred. As the average length of BT sequences in the PBST and PBAT copolyesters is very small (close to two, Table 1), the polymers lack enough chain regularity, so there might be a lot of defects in the formed crystals. Possibly for this reason, it is difficult to form a sufficiently ordered crystal structure with a big enough size. In fact, it was reported that the spherulite size of PBST_50_ determined by light scattering pattern is only 11.4 um in diameter [35].

### 3.4. Tensile Properties

**Figure 5:**
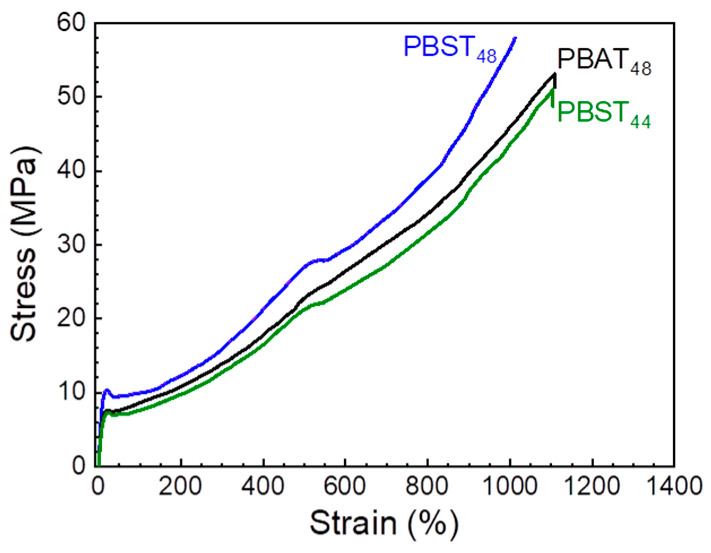
Tensile stress-strain curves of PBAT_48_, PBST_48,_ and PBST_44_.

The tensile properties of PBAT_48_, PBST_48,_ and PBST_44_ were further investigated. Typical tensile curves are shown in Figure 5. As semi-crystalline polymers, they all exhibited typical ductile-tensile behavior, displaying elastic deformation, yielding, high elastic deformation, and strain hardening in sequence with increasing tensile strain. In comparison with PBAT_48_, PBST_48_ displayed the same tensile stress at break but a clearly higher tensile modulus (*E*, 144 MPa vs. 107 MPa) and yield strength (*σ_y_*, 9.9 MPa vs. 7.7 MPa) because of its lower chain flexibility and higher crystallinity, as demonstrated by the nonisothermal melt crystallization results. PBST_44_ has less BT molar content but the same BT mass content and similar crystallinity to PBAT_48_, so it exhibited *E* and *σ_y_* comparable to PBAT_48_, and slightly lower tensile stress at break (49 MPa vs. 55 MPa). As PBAT is too soft for many applications, the higher modulus and yielding strength of the PBST copolyesters are very desirable properties for practical applications. In addition, when compared with commercial PBAT reported in the literature [37], the PBST copolyesters exhibited not only a much higher modulus (107–144 MPa vs. 46 MPa [37]), but also a much higher strength (49–56 MPa vs. 29 MPa [37]). This result also supports the conclusion that the PBST copolyesters used in this study have a sufficiently high molecular weight. 

## 4. Conclusions

In this study, the melt crystallization of PBST_48_ and PBST_44_ was investigated in nonisothermal and isothermal manners and compared with PBAT_48_. PBST_48_ showed a PBAT_48_-comparable melt crystallization performance at a conventional DSC cooling rate of 10 °C/min or at isothermal conditions, but showed a clearly lower melt crystallization temperature and enthalpy and a much longer crystallization time at a cooling rate of 40 °C/min. PBST_44_, which has the same mass percentage of BT unit as PBAT_48_, not only showed a much slower crystallization rate at a cooling rate of 10 °C/min, but it also completely lost its melt crystallization ability at a cooling rate of 40 °C/min. The weaker melt crystallization ability of PBSTs at the same copolymer composition is attributed to the shorter aliphatic diacid moiety in PBST than in PBAT (succinate vs. adipate) and the resultant weaker chain mobility. It is the main reason why PBST is more difficult to pelletize and injection-mold. Due to the difference in melt crystallization at rapid cooling and chain flexibility, the PBST films show higher light transmittance, and PBST_48_ showed an obviously higher tensile modulus and yielding strength than PBAT_48_. Although PBST crystallizes more slowly under rapid cooling, it has superior mechanical, optical, and gas barrier properties to PBAT and therefore appears to be a more promising biodegradable aliphatic-aromatic copolyester when it comes to more demanding applications, such as food packaging. The promotion of the melt crystallization of PBST under rapid cooling will be reported later.

## Figures and Tables

**Table 1:** Structural characteristics of PBAT_48_, PBST_44_, and PBST_48_ used in this study.

Sample	*φ*_TPA_ ^a^ (mol%)	*ϕ*_BT_ ^b^ (mol%)	*ϕ*_w,BT_ ^c^ (wt%)	*L*_n,BX_ ^d^	*L*_n,BT_ ^e^	*R* ^f^	*IV* ^g^ (dL/g)
PBAT_48_	46.0	47.8	50.2	2.07	1.89	1.01	1.15
PBST_48_	46.0	48.4	54.5	2.03	1.97	1.00	1.21
PBST_44_	42.0	44.4	50.5	2.24	1.81	1.00	1.08

^a^ Molar percentage of TPA in diacid feed; ^b^ molar percentage of BT unit in copolyesters, calculated from ^1^H NMR results; ^c^ mass percentage of BT unit in copolyesters calculated from *ϕ*_BT;_ ^d,e^ number-average length of BX (X = A or S) and BT sequences; ^f^ degree of randomness; and ^g^ intrinsic viscosity measured at 25 °C using chloroform as solvent.

**Table 2:** Thermal transition properties of PBAT4_8_, PBST_48_, and PBST_44_ during cooling and 2nd heating DSC scans ^a.^.

Sample	*T*_c_ ^b^ (°C)	Δ*H*_c_ ^c^ (J/g)	*t*_1/2_ ^d^ (s)	*T*_g_ (°C)	*T*_cc_ (°C)	Δ*H*_cc_ ^e^ (J/g)	*T*_m_ (°C)	Δ*H*_m_ ^f^ (J/g)
	Cooling at 10 °C/min			2nd heating at 10 °C/min				
PBAT_48_	78	19.8	43	−31	nd	nd	122	19.2
PBST_48_	78	20.4	45	−13	nd	nd	128	19.7
PBST_44_	47	17.3	98	−16	23	1.2	112	18.8
PBAT ^g^	82, 80	8.3, 13.1	/	−33, −35	/	/	120, 119	5.7, 8.5
		Cooling at 40 °C/min		2nd heating at 10 °C/min				
PBAT_48_	63	20.1	15	−31	nd	nd	118	20.6
PBST_48_	51	16.9	30	−14	21	4.7	127	21.6
PBST_44_	nd	nd	nd	−5	24	15.4	111	19.2

^a^: Heat history was erased at 220 °C for 5 min; ^b^ melt crystallization temperature; ^c^ melt crystallization enthalpy; ^d^ melt crystallization half period—namely, the time taken for the relative crystallinity reaching 50%; ^e^ cold crystallization enthalpy during 2nd heating; ^f^ melting enthalpy during 2nd heating; ^g^ commercial PBAT with *M*_n_ of 39,700 g/mol and melt flow index 3.5 g/10 min, reported in ref. [37,38].

**Table 3:** Isothermal melt crystallization kinetic constants BT sequence of PBAT_48_, PBST_48_ and PBST_44_ *.

Sample	*T*_c_ (°C)	*n*	*K* (min^−*n*^)	*t*_1/2_ (min)
PBAT_48_	78	3.2	3.3	0.61
PBST_48_	78	3.2	3.3	0.61
PBST_44_	47	3.1	2.3	0.68

* *K* and *n* are calculated from the data in the time range from 0.05 to 0.22 min, as indicated by the grey area in Figure 4a,c.

**Table 4:** Melting point (*T*_m_) and melting enthalpies (Δ*H*_m_) of PBAT_48_, PBST_48_, and PBST_44_ during 2nd heating after isothermal crystallization.

Sample	*T*_m1_ (°C)	Δ*H*_m1_ (J/g)	*T*_m2_ (°C)	Δ*H*_m2_ (J/g)	Δ*H*_m,sum_^a^ (J/g)
PBAT_48_	88	2.6	123	16.2	18.8
PBST_48_	88	2.4	130	19.5	21.9
PBST_44_	59	1.9	115	18.2	20.1

^a^: Total enthalpy of melting, Δ*H*_m,sum_ = Δ*H*_m1_ + Δ*H*_m2_.

**Table 5:** Haze, transmittance, and tensile properties of PBAT_48_, PBST_48_ and PBST_44_.

Sample	Haze (%)	Trans (%)	*E* ^a^ (MPa)	*σ_y_* ^b^ (MPa)	*σ_b_* ^c^ (MPa)	*ε_y_* ^d^ (%)	*ε_b_* ^e^ (%)
PBAT_48_	98.4	45.0	107 ± 1	7.7 ± 0.1	55 ± 1	19.8 ± 0.1	1110 ± 10
PBST_48_	98.7	51.3	144 ± 5	9.9 ± 0.2	56 ± 2	19.3 ± 0.4	995 ± 12
PBST_44_	98.5	53.7	97 ± 1	7.5 ± 0.2	49 ± 3	22.4 ± 0.3	1040 ± 42
PBAT ^f^	/	/	46 ± 6	/	29 ± 4	/	655 ± 65

^a^ Tensile modulus; ^b^ stress at yielding; ^c^ stress at break; ^d^ strain at yielding; ^e^ strain at break; and ^f^ commercial PBAT with *M*_n_ of 39,700 g/mol and melt flow index 3.5 g/10 min, reported in ref. [37].

## Data Availability

The original contributions presented in the study are included in the article/supplementary material, further inquiries can be directed to the corresponding author.

## Supplementary Materials

The following supporting information can be downloaded at: https://www.mdpi.com/article/10.3390/polym16172445/s1, Figure S1: ^1^H NMR spectra (solvent: CDCl_3_) of PBAT_48_, PBST_48_ and PBST_44_, Figure S2: (a) First heating DSC scan (10 ^o^C/min) curves and (b) WAXD patterns of PBAT_48_, PBST_48_ and PBST_44_.
