# Supplementary material for: A Comparative Study on the Melt Crystallization of Biodegradable Poly(butylene succinate-co-terephthalate) and Poly(butylene adipate-co-terephthalate) Copolyesters
Source: Polymers (Basel). 2024 Aug 29;16(17):2445. doi: 10.3390/polym16172445 (PMC11397942; doi:10.3390/polym16172445)
Supplement: Supplementary file 1 [file polymers-16-02445-s001.zip › polymers-3160786-supplementary.pdf]

Supporting information

A Comparative Study on the Melt Crystallization of Biodegradable Poly(butylene succinate-*co*-terephthalate) and Poly(butylene adipate-*co*-terephthalate) Copolyesters

Pengkai Qin [1,2] and Linbo Wu [1,2,*]

[1] Key Laboratory of Biomass Chemical Engineering of Ministry of Education, College of Chemical and Biological Engineering, Zhejiang University, Hangzhou 310058, China; qinpk@zju.edu.cn

[2] State Key Laboratory of Chemical Engineering, College of Chemical and Biological Engineering, Zhejiang University, Hangzhou 310058, China

* Correspondence: wulinbo@zju.edu.cn

1. ^{1}H NMR characterization and calculation of chain structure parameters

The chemical structure of $PBAT_{48}$, $PBST_{48}$ and $PBST_{44}$ was characterized with ^{1}H NMR. **Figure S1** shows the ^{1}H NMR spectra ($CDCl_3$ as solvent) and the attribution of the chemical shift. In comparison with the chemical shift of ester bond-neighboring C**H**$_2$ (a) in the adipate moiety in $PBAT_{48}$, that in the succinate moiety in $PBST_{48}$ and $PBST_{44}$ moves to the higher field. It should be noted that signal **b** is observed only in $PBAT_{48}$. Because of the difference in chemical environment, the chemical shifts of the ester bond-neighboring C**H**$_2$ (c) and the secondary C**H**$_2$ (d) in BDO residue split into four peaks (**c**$_{1\text{-}4}$, **d**$_{1\text{-}4}$).

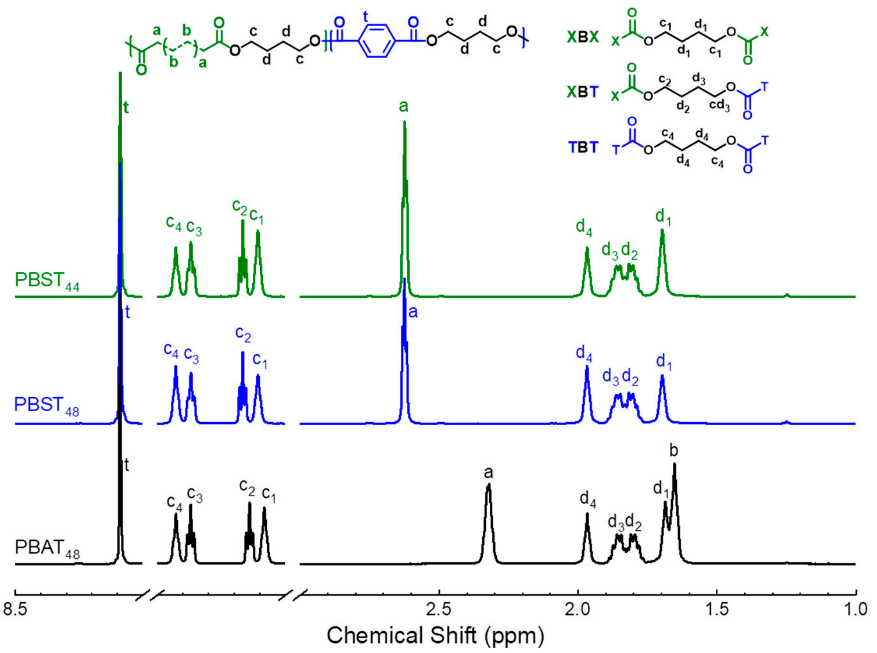

Figure S1. ^{1}H NMR spectra (solvent: $CDCl_3$) of $PBAT_{48}$, $PBST_{48}$ and $PBST_{44}$.

The copolymer molar composition (ϕ_{BT}) is defined as the molar fraction of butylene terephthalate (BT) repeat unit in the copolymer. It is calculated by Equation (S1), in which A_t and A_a are the areas of peak **t** and **a** respectively. The copolymer mass composition ($\phi_{w,BT}$) is defined as the mass fraction of BT unit. It is calculated from ϕ_{BT} and the molecular weight of BT and BX units (M_{BT}, $M_{BX,\ X=A\ or\ S}$), see Equation (S2). From the peak areas of **c1-4**, the number-average sequence length of BX and BT units ($L_{n,BX}$ and $L_{n,BT}$) and the degree of randomness (R) are calculated with Equations (S3)–(S5).

$$\phi_{\mathrm{BT}} = \frac{A_{\mathrm{t}}}{A_{\mathrm{t}} + A_{\mathrm{a}}} \tag{S1}$$

$$\phi_{\mathrm{m,BT}} = \frac{\phi_{\mathrm{BT}} M_{\mathrm{BT}}}{\phi_{\mathrm{BT}} M_{\mathrm{BT}} + (1 - \phi_{\mathrm{BT}}) M_{\mathrm{BX}}} \tag{S2}$$

$$L_{\mathrm{BX}} = 1 + \frac{2A_{\mathrm{c_1}}}{A_{\mathrm{c_2}} + A_{\mathrm{c_3}}} \tag{S3}$$

$$L_{\mathrm{BT}} = 1 + \frac{2A_{\mathrm{c_4}}}{A_{\mathrm{c_2}} + A_{\mathrm{c_3}}} \tag{S4}$$

$$R = 1/L_{\mathrm{BX}} + 1/L_{\mathrm{BT}} \tag{S5}$$

2. First heating DSC curves and WAXD patterns

The first heating DSC curves at 10 ^{o}C/min and WAXD patterns of $\mathrm{PBAT_{48}}$, $\mathrm{PBST_{48}}$ and $\mathrm{PBST_{44}}$ are shown in **Figure S2**. Two melting peaks were observed for all the copolyesters. The higher temperature peak is the major one and is attributed to the melting of BT crystal, and the lower temperature one is attributed to the melting of BX (X=A or S) crystal. It means that both BA or BS and BT sequences can crystallize at a copolymer composition close to that of commercial PBAT (ϕ_{BT} ~48 mol% or $\phi_{\mathrm{w,BT}}$ ~50 wt%). But only the diffraction peaks of BT crystal (2θ angle 16.0 ^{o}, 17.4 ^{o}, 20.3 ^{o}, 23.2 ^{o}, 25.0 ^{o}) can be observed in the WAXD patterns (**Figure S2b**). These results are similar to those reported previously for $\mathrm{PBST_{50}}$[35].

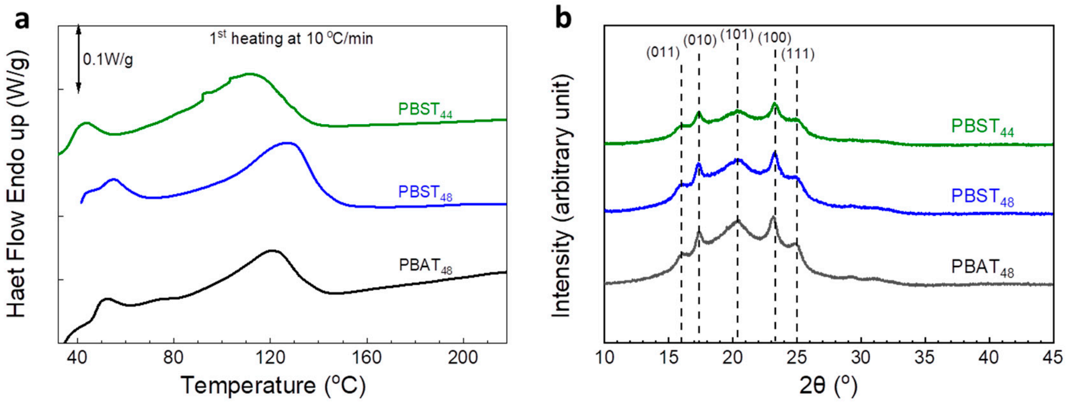

Figure S2 a) First heating DSC scan (10 °C/min) curves and **b**) WAXD patterns of $PBAT_{48}$, $PBST_{48}$ and $PBST_{44}$.
